# The spatial landscape of the peritumoral interface: ARG1^+^ PMN-MDSCs reduction and NETosis as indicators of occult metastasis in tongue squamous cell carcinoma

**DOI:** 10.3389/fimmu.2026.1910935

**Published:** 2026-08-19

**Authors:** Shengqi Zhang, Jiaye Xu, Chunyu Zhang, Xuefen Li, Diancan Wang, Xiangjun Li, Chuanbin Guo, Lin Wang

**Affiliations:** 1 Department of Oral and Maxillofacial Surgery, Peking University School and Hospital of Stomatology, Beijing, China; 2 Peking University School and Hospital of Stomatology & National Center for Stomatology & National Clinical Research Center for Oral Diseases & National Engineering Research Center of Oral Biomaterials and Digital Medical Devices & Beijing Key Laboratory of Digital Stomatology & NHC Key Laboratory of Digital Stomatology & NMPA Key Laboratory for Dental Materials, Beijing, China; 3 Department of Oral and Maxillofacial Surgery, School and Hospital of Stomatology, Hebei Medical University, Hebei Technology Innovation Center of Oral Health, Key Laboratory of Stomatology and Clinical Research Centre for Oral Diseases, Shijiazhuang, Hebei, China; 4 Central Laboratory, Peking University School and Hospital of Stomatology, Beijing, China

**Keywords:** Arginase-1, neutrophil extracellular traps (NETs), occult metastasis, PMN-MDSCs, spatial immunology, tongue squamous cell carcinoma

## Abstract

**Background:**

Predicting occult lymph node metastasis in early-stage tongue squamous cell carcinoma remains a clinical challenge because traditional markers, such as depth of invasion, exhibit limited accuracy. This study aimed to identify spatial immunological biomarkers to improve risk stratification and clinical decision-making.

**Methods:**

Olink proteomic profiling was conducted to screen for metastasis-associated biomarkers. Subsequently, multiplex immunofluorescence coupled with artificial intelligence-powered spatial analysis was utilized to evaluate the microenvironment in primary lesions and paired cervical lymph nodes. A novel “Spatial Availability Index” was applied to map cellular distribution, and diagnostic performance was evaluated using receiver operating characteristic curve analysis.

**Results:**

Arginase-1 (ARG1) was identified as a top candidate biomarker, exhibiting a unique “tissue-low, serum-high” expression pattern in cases with occult metastasis. Spatial analysis revealed a significant depletion of ARG1^+^ polymorphonuclear myeloid-derived suppressor cells (PMN-MDSCs) in both primary tumors and paired lymph nodes during metastatic progression. Specifically, the reduction of these intact cells was most pronounced within a 35-μm proximal segment from the tumor boundary. This localized cellular loss inversely correlated with an accumulation of neutrophil extracellular traps (NETs) at the peritumoral interface, indicating that ARG1^+^ PMN-MDSC-associated spatial alterations coincide with NET-associated remodeling at the peritumoral interface. Integrating the intrastromal density of ARG1^+^ PMN-MDSCs with depth of invasion significantly enhanced the diagnostic accuracy for occult metastasis (Area Under the Curve = 0.8318) compared to using depth of invasion alone.

**Conclusions:**

Early metastatic risk is driven by localized alterations within the myeloid compartment, characterized by the transition of intact ARG1^+^ PMN-MDSCs to NETs within a 35-μm peritumoral hotspot. This spatial biomarker provides a high-resolution tool to optimize patient selection for elective neck dissection.

## 1 Introduction

Cancers of the lip and oral cavity represent a significant global health burden, with Oral squamous cell carcinoma (OSCC) accounting for over 90% of cases, and tongue squamous cell carcinoma (TSCC) being the most prevalent and aggressive subtype (1–4). The prognosis of TSCC is dictated primarily by the status of cervical lymph node metastasis, where the transition to nodal involvement results in a staggering 50% reduction in overall survival (5). In early-stage (cT1-2N0) TSCC, the clinical management of the neck remains highly controversial. Current clinical consensus favors elective neck dissection (END) over ‘watchful waiting’ due to its superior survival benefit (6), particularly recommending the procedure for cases with a depth of invasion (DOI) ≥ 4 mm to address potential occult metastasis (7–9). However, the procedure is fraught with significant morbidity, including nerve injury and shoulder dysfunction, which profoundly impacts patient quality of life (10–12). This underscores an urgent need for reliable predictive biomarkers to accurately identify occult metastasis.

Recent advances have redefined regional lymph nodes (LNs) from passive anatomical filters into essential hubs for immune surveillance and the initiation of systemic anti-tumor responses (13–16). Emerging evidence suggests that the primary tumor actively orchestrates the formation of a pre-metastatic niche within these sentinel LNs long before physical dissemination occurs (17–19). Among the myriad of immune regulators driving this metastatic priming, polymorphonuclear myeloid-derived suppressor cells (PMN-MDSCs) have emerged as important regulators of tumor immune suppression, partly through the secretion of Arginase-1 (ARG1), which can impair local T-cell responses (17, 20–22). Furthermore, these pathologically activated PMN-MDSCs can undergo NETosis, extruding neutrophil extracellular traps (NETs) that create dense physical barriers to shield cancer cells and facilitate metastatic escape (23–27). Despite these advances, whether spatial alterations of ARG1^+^ PMN-MDSCs can serve as biomarkers for occult lymph node metastasis remains unclear. In particular, the relationship between the spatial distribution of intact ARG1^+^ PMN-MDSCs, NET-associated changes, and metastatic risk has not been fully elucidated in TSCC.

In this study, we aimed to identify and evaluate spatial immune biomarkers associated with occult lymph node metastasis in early-stage TSCC. To address this gap, we employed Olink proteomic profiling (28) and multiplexed immunofluorescence (mIF), integrated with digital spatial analysis to map the immune landscape of the TSCC microenvironment. Specifically, we investigated the expression pattern, spatial distribution, and clinical relevance of ARG1^+^ PMN-MDSCs in primary tumors and lymph nodes, and assessed their potential value in predicting occult lymph node metastasis. Furthermore, we explored the biological context underlying these spatial immune alterations by examining their association with NET-associated signals at the tumor-stroma interface. By integrating spatial immune parameters with established clinicopathological factors, we developed a spatio-clinical model for risk stratification of early-stage TSCC. Our findings provide a spatially resolved immune framework for understanding occult metastatic potential and highlight ARG1^+^ PMN-MDSCs as candidate biomarkers for further clinical validation.

## 2 Methods

### 2.1 Patients cohort and study design

We retrospectively analyzed 113 consecutive patients with clinical T1-2N0M0 (early-stage) primary TSCC treated at the Department of Oral and Maxillofacial Surgery, Peking University School and Hospital of Stomatology between 2014 and 2017 (**Table 1**). Patients were stratified based on pathological nodal status into two groups: the occult lymph node metastasis (OLNM) group (n = 33) and the non-metastasis (NM) group (n = 80). Occult metastasis was defined as the presence of pathologically confirmed metastatic foci in elective neck dissection specimens despite a clinically negative neck (cN0) prior to surgery. Inclusion criteria were (1): Pathologically confirmed TSCC with DOI re-evaluation according to AJCC 8th (29) (2); Clinical tumor stage T1 or T2 (3). cN0 status, defined by both physical examination and preoperative imaging (contrast-enhanced CT) showing no palpable nodes or lymph nodes with a short-axis diameter <10 mm, absent of central necrosis or extracapsular spread (4); All patients underwent radical primary tumor resection combined with elective neck dissection. Exclusion criteria included prior therapy, recurrent cases, inadequate specimens.

**Table 1:** Baseline characteristics of the TSCC patients.

Characteristics	OLNM group	NM group	*P*
	Retrospective cohort		
	n=33	n=80	
Age (mean ± SD)	55.73 ± 12.78	53.18 ± 12.88	0.339
Gender			0.532
Male	22(66.67%)	48(60.00%)	
Female	11(33.33%)	32(40.00%)	
Smoking history			0.674
Yes	12(36.36%)	34(42.50%)	
No	21(63.64%)	46(57.50%)	
Alcohol history			0.656
Yes	11(33.33%)	23(28.75%)	
No	22(66.67%)	57(71.25%)	
cT grade			0.046*
T1	5(15.15%)	27(33.75%)	
T2	28(84.85%)	53(66.25%)	
DOI (mm)	6.11 ± 2.75	3.97 ± 2.35	<0.001***
DOI status, n%			0.012*
≥4mm	23(69.70%)	35(43.75%)	
<4mm	10(30.30%)	45(56.25%)	
Differentiation			0.012*
Well	6(18.18%)	38(47.50%)	
Moderate	18(54.55%)	28(35.00%)	
Poor	9(27.27%)	14(17.50%)	
Growth pattern			0.016*
Exophytic	3(9.10%)	21(26.25%)	
Infiltrative	10(30.30%)	33(41.25%)	
Ulcerative	20(60.60%)	26(32.50%)	

Data are shown as mean ± SD or n (%). Continuous variables were compared using unpaired t-tests, and categorical variables using Chi-square or Fisher’s exact tests. *P < 0.05, **P < 0.01, ***P < 0.001. TSCC, tongue squamous cell carcinoma; NM, non-metastasis; OLNM, occult lymph node metastasis; DOI, depth of invasion.

Due to the retrospective nature of the tissue cohort and differences in specimen availability, multiple nested sub-cohorts were generated from the retrospective tissue cohort for specific experimental analyses. Propensity score matching (PSM) was performed separately for each comparative analysis to minimize baseline imbalance between OLNM and NM groups. Age and sex were selected as predefined demographic covariates for PSM. Other available clinicopathological variables were evaluated before and after matching to assess baseline comparability between groups. The caliper width was set at 0.2 times the standard deviation of the propensity score logit.

#### 2.1.1 Tumor tissue Olink proteomic sub-cohort

Among the retrospective tissue cohort, fresh-frozen (FF) primary tumor tissues were available from 55 patients (OLNM: n = 21; NM: n = 34) and stored at −80 °C. Because fresh-frozen specimens were only available from a subset of patients, tumor tissue Olink proteomic profiling was performed in this subgroup. After 1:1 PSM, 17 matched pairs (34 patients) were selected for Olink proteomic analysis (**Supplementary Table 1**).

#### 2.1.2 Spatial validation sub-cohort

For spatial immune profiling, FFPE primary tumor tissues and corresponding cervical lymph node specimens were available from the retrospective tissue cohort. Following an independent 1:1 PSM procedure, 23 matched pairs (46 patients) were selected for multiplex immunofluorescence (mIF) staining and digital spatial analysis (**Supplementary Table 2**).

#### 2.1.3 NETosis mechanistic validation sub-cohort

To further investigate the relationship between ARG1^+^ PMN-MDSCs and NETosis, 24 primary TSCC sections (OLNM: n = 12; NM: n = 12) were selected from the spatial validation sub-cohort for high-resolution NETosis imaging. This mechanistic sub-cohort was established to characterize NETosis-associated features and maintained balanced representation of OLNM and NM cases.

#### 2.1.4 Independent serum proteomic cohort

In addition, an independent serum cohort consisting of 17 TSCC patients (OLNM: n = 7; NM: n = 10) was prospectively collected for preoperative serum Olink proteomic profiling. This serum cohort was independent from the retrospective tissue cohort and was used to evaluate systemic alterations of candidate biomarkers.

The establishment of each experimental sub-cohort is summarized in **Supplementary Figure 1**.

### 2.2 Multiplexed proteomic profiling (Olink PEA)

The Olink Target 96 Immuno-Oncology panel (92 biomarkers) was used to profile fresh-frozen primary tumors (n=34) and serum (n=17). Fresh-frozen tumor specimens were collected immediately after surgical resection, stored at −80 °C, and shipped on dry ice to Olink Proteomics for sample processing and analysis. Preoperative peripheral blood samples were collected from patients, and serum was obtained by centrifugation and stored at −80 °C until shipment to Olink Proteomics. Tissue homogenization, protein extraction, serum sample preparation, and subsequent proteomic profiling were performed by Olink according to the manufacturer’s standardized protocol. Quantification was performed according to the Proximity Extension Assay (PEA) workflow (30). Data were processed using Olink NPX Manager; Normalized Protein Expression (NPX) values were used for downstream analysis. Olink assay data were quality controlled and normalized according to the manufacturer’s standard workflow using internal controls and inter-plate controls to minimize technical variation. NPX values represent normalized protein expression on a log2 scale, with higher NPX values indicating higher protein abundance. The complete list of proteins included in the Olink Target 96 Immuno-Oncology panel analyzed in this study is provided in **Supplementary Table 3**.

### 2.3 Bioinformatics analysis

The final NPX dataset provided after Olink quality control was used for downstream analysis. No additional imputation of missing values or manual outlier removal was required before downstream analysis. All samples were processed simultaneously using the same Olink panel and experimental workflow, and therefore no separate analytical batches were introduced. Differential protein analysis between OLNM and NM groups was performed using the limma R package based on NPX values. Proteins with nominal P < 0.05 were considered candidates for subsequent biomarker prioritization, given the exploratory nature of the discovery analysis and subsequent validation using multiplex immunofluorescence. Candidate biomarkers were prioritized via Random Forest ranking (Mean Decrease Accuracy/Gini) and validated by ROC/AUC analysis. Functional enrichment was performed through Gene Ontology (GO) and Kyoto Encyclopedia of Genes and Genomes (KEGG) mapping to decode biological pathways. Significantly enriched pathways were identified using a hypergeometric test, with the entire Olink Immuno-Oncology panel serving as the background reference. Visualization of the proteomic landscape, including volcano plots, heatmaps, and enrichment networks, was generated using the ggplot2 and pheatmap R packages.

### 2.4 Multiplex immunofluorescence staining

FFPE sections from the Validation Cohort (*n* = 46, 23 matched pairs) and the Independent Mechanistic Set (*n* = 24) were processed for mIF staining using the Opal™ 7-Color Kit (Akoya).

Briefly, slides underwent deparaffinization, rehydration, and heat-induced antigen retrieval (HIER). Each staining cycle consisted of (1): protein blocking (2), primary antibody incubation (3), horseradish peroxidase (HRP)-conjugated secondary antibody incubation, and (4) signal amplification with tyramine-conjugated fluorophores (Opal 480, 520, 570, 620, 690, and 780). After six rounds of staining, nuclei were counterstained with spectral DAPI.

Two panels were optimized: Spatial architecture panel, designed for primary tumor and lymph node mapping, including Pan-CK(1:200, ZM-0069, ZSGB-BIO), CD11b (1:200, #49420, CST), CD14 (1:400, ab183322, Abcam), CD15(1:200, ab135377, Abcam), HLA-DR (1:1000, ab92511, Abcam) and ARG1(1:500, ab133543, Abcam). Mechanistic NETosis panel, designed for the independent validation set to characterize functional myeloid states at the interface, including PanCK (1:200), CD11b (1:200), CD15 (1:200), ARG1 (1:500), CitH3 (1:500, ab5103, Abcam) and MPO (1:1000, ab208670, Abcam).

### 2.5 Multispectral imaging and digital spatial analysis

Whole-slide images were acquired using the Vectra Polari multispectral imaging system (Akoya Biosciences). To ensure signal fidelity, multispectral images were spectrally unmixed using inForm software (v2.4.8, Akoya Biosciences) to eliminate autofluorescence and crosstalk between overlapping fluorophores. For image analysis, tumor-containing regions were manually annotated as regions of interest (ROIs) based on PanCK expression and histopathological morphology. Instead of selecting representative fields of view, all evaluable tumor areas within each section were included for downstream quantitative analysis. The number and size of ROIs varied among individual specimens due to differences in tumor area, tissue architecture, and sample availability. Areas with tissue artifacts, including folds, detachment, necrosis, or insufficient staining quality, were excluded. AI-powered tissue and single-cell segmentation were performed using HALO (v4.1.5944). Following spectral unmixing and autofluorescence correction, individual cells were segmented based on DAPI nuclear staining combined with cellular marker signals. Fluorescence intensity of each marker was quantified at the single-cell level. Marker positivity was determined according to fixed fluorescence intensity thresholds, and identical thresholds and analysis parameters were applied across all samples. Specific cell populations were classified based on a combinatorial marker panel. Based on previously established criteria for human PMN-MDSCs, cells exhibiting a CD11b^+^CD15^+^CD14^-^HLA-DR^-^ phenotype with ARG1 expression were operationally defined as ARG1^+^ PMN-MDSCs in this study (31). Cell densities (cells/mm²) were quantified within the parenchymal and stromal compartments separately.

### 2.6 Spatial proximity and neighborhood analysis

To investigate the spatial architecture of the tumor microenvironment, we performed proximity analysis using the HALO Spatial Analysis module. The mean distance from each PMN-MDSC to the nearest PanCK^+^ tumor cell was calculated. Furthermore, we implemented a segmental spatial analysis (Spatial Availability Index) to quantify PMN-MDSC abundance at 5μm intervals from the tumor boundary (0–100μm), thereby mapping the localized infiltration patterns associated with occult metastasis.

### 2.7 Quantitative analysis of NETs

To specifically characterize the formation of NETs at the tumor-stroma interface, digital image analysis was performed using QuPath (v0.3.0). NETs were identified by the co-localization of extracellular CitH3 and MPO signals. The percentage of NETs-covered area (NETs%) was quantified within discrete distance intervals from the tumor boundary.

### 2.8 Statistical analysis

Continuous variables were analyzed using the unpaired two-tailed Student’s t-test for independent groups. Comparisons involving variables across multiple intervals were performed via two-way ANOVA followed by Sidak’s *post-hoc* tests. Categorical variables were compared using the Chi-square test or Fisher’s exact test, as appropriate. The associations between experimental variables were evaluated using Pearson correlation analysis, and the diagnostic performance of relevant clinical and immunological parameters was assessed by ROC curve analysis and the AUC. Overall survival (OS), defined as the duration from the date of primary surgery to the date of death or last follow-up, was estimated using the Kaplan-Meier method and compared via the log-rank test. All statistical analyses were conducted using GraphPad Prism 10 and R software (version 4.2.2). All tests were two-sided, and significance levels were defined as *P < 0.05, **P < 0.01, and ***P < 0.001.

## 3 Results

### 3.1 Clinical and pathological landscape identifies a diagnostic gap in early-stage TSCC

To delineate the clinical landscape associated with early-stage TSCC, we analyzed a comprehensive retrospective cohort (n=113), whose clinicopathological characteristics are summarized in **Table 1**.

**Figure 1:**
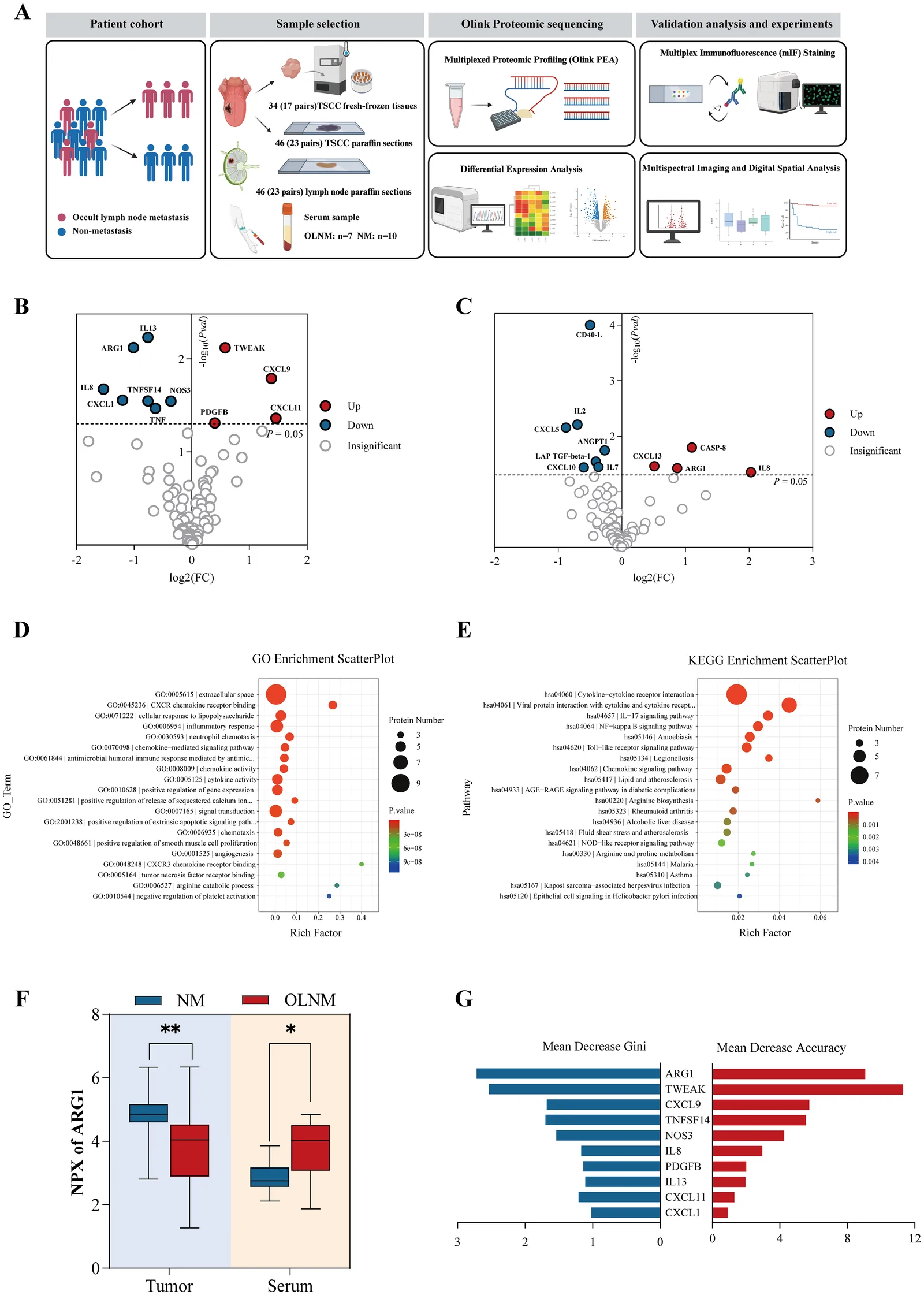
Systematic screening identifies a divergent tissue-serum expression pattern of ARG1. **(A)** Schematic representation of the experimental workflow. Created with https://www.BioRender.com. **(B, C)** Volcano plots displaying differentially expressed proteins (DEPs) in primary tumor tissues **(B)** and preoperative serum **(C)** between the NM and OLNM groups. **(D, E)** Gene Ontology (GO) **(D)** and Kyoto Encyclopedia of Genes and Genomes (KEGG) **(E)** enrichment analyses of the identified DEPs from primary lesions. **(F)** Box plots showing the divergent expression pattern of ARG1 in tissue versus serum. **(G)** Feature importance ranking derived from the Random Forest algorithm. *P < 0.05, **P < 0.01, ***P < 0.001. NM, non-metastasis; OLNM, occult lymph node metastasis; mIF, multiplex immunofluorescence. DEPs, differentially expressed proteins. ARG1, arginase-1.

In the initial comparison, no significant differences were observed in demographic characteristics, including age, sex, smoking history, and alcohol consumption, between OLNM and NM groups (P > 0.05). In contrast, several pathological parameters were associated with OLNM status. Specifically, the OLNM group exhibited a higher proportion of cT2 tumors compared with the NM group (84.85% vs. 66.25%, P = 0.046). In addition, increased DOI, poorer tumor differentiation, and more aggressive growth patterns were also observed in patients with OLNM. However, despite their clinical relevance, these conventional pathological parameters showed incomplete separation between OLNM and NM groups, indicating that additional biological markers may be required for more precise risk stratification. Notably, among patients traditionally categorized as low-risk based on DOI (<4 mm), 10/55 patients (18.18%) still harbored occult lymph node metastasis (**Table 1**). This limitation of morphology-based risk assessment highlights the need for higher-resolution immunological biomarkers. To identify molecular features associated with these clinically occult metastatic events, nested sub-cohorts were subsequently established from the retrospective tissue cohort according to specimen availability and experimental objectives. Propensity score matching (PSM) was applied to minimize potential baseline imbalance, followed by tumor tissue proteomic profiling and spatial multiplex immunofluorescence analysis (**Figure 1A**).

### 3.2 Proteomic profiling identifies a divergent pattern of ARG1 across systemic and local discovery cohorts

To identify potential biomarkers associated with OLNM, we profiled 92 immuno-oncology-related proteins using the Olink Proximity Extension Assay. In the primary tumor tissues, 11 differentially DEPs were identified between the OLNM and NM groups. Specifically, four proteins—CXCL9, CXCL11, PDGFB, and TWEAK—were significantly upregulated in the OLNM group. Conversely, seven proteins exhibited significant downregulation, with ARG1, IL-8, CXCL1, TNF, and IL-13 showing the most pronounced reductions (**Figure 1B**, **Supplementary Figure 2A**).

To evaluate the functional significance of these proteomic shifts, we performed GO and KEGG enrichment analyses (**Figures 1D, E**). The identified DEPs localized predominantly to the extracellular space and participated in chemokine receptor binding, NF-kappa B, and Toll-like receptor signaling pathways. Notably, several of the most significantly downregulated markers, including ARG1, IL-8, and CXCL1, are established mediators of myeloid cell recruitment and inflammatory signaling. This coordinated reduction highlights a significant shift in the myeloid-associated immune environment within OLNM primary tumors.

Intriguingly, in systemic discovery cohort, serum proteomic analysis revealed a divergent expression pattern for these markers. In contrast to their depletion in the primary tumor tissue, ARG1 and IL-8 were significantly elevated in the preoperative serum of patients with OLNM (**Figures 1C, F**, **Supplementary Figure 2B**). This “Tissue-Low, Serum-High” discrepancy defines a distinct protein distribution pattern between the localized tumor site and the systemic circulation.

To prioritize the most robust predictors among the tissue-derived DEPs, we applied a Random Forest algorithm and ROC curve analysis. ARG1 and TWEAK emerged as the top-ranking features, with TWEAK ranking first in Mean Decrease Accuracy and ARG1 achieving the highest Mean Decrease Gini (**Figure 1G**). ROC analysis further demonstrated that ARG1 exhibited superior individual discriminative performance (AUC = 0.80) compared with TWEAK (AUC = 0.75, **Supplementary Figure 2C**). Considering the additional tissue–serum discordant expression pattern of ARG1 and its potential for spatial identification of ARG1^+^ immune populations, ARG1 was selected as the central candidate for subsequent spatial investigation.

### 3.3 Compartment-specific alterations of ARG1^+^ PMN-MDSCs in both primary lesions and paired lymph nodes

#### 3.3.1 Phenotypic characterization of ARG1^+^ populations

**Figure 2:**
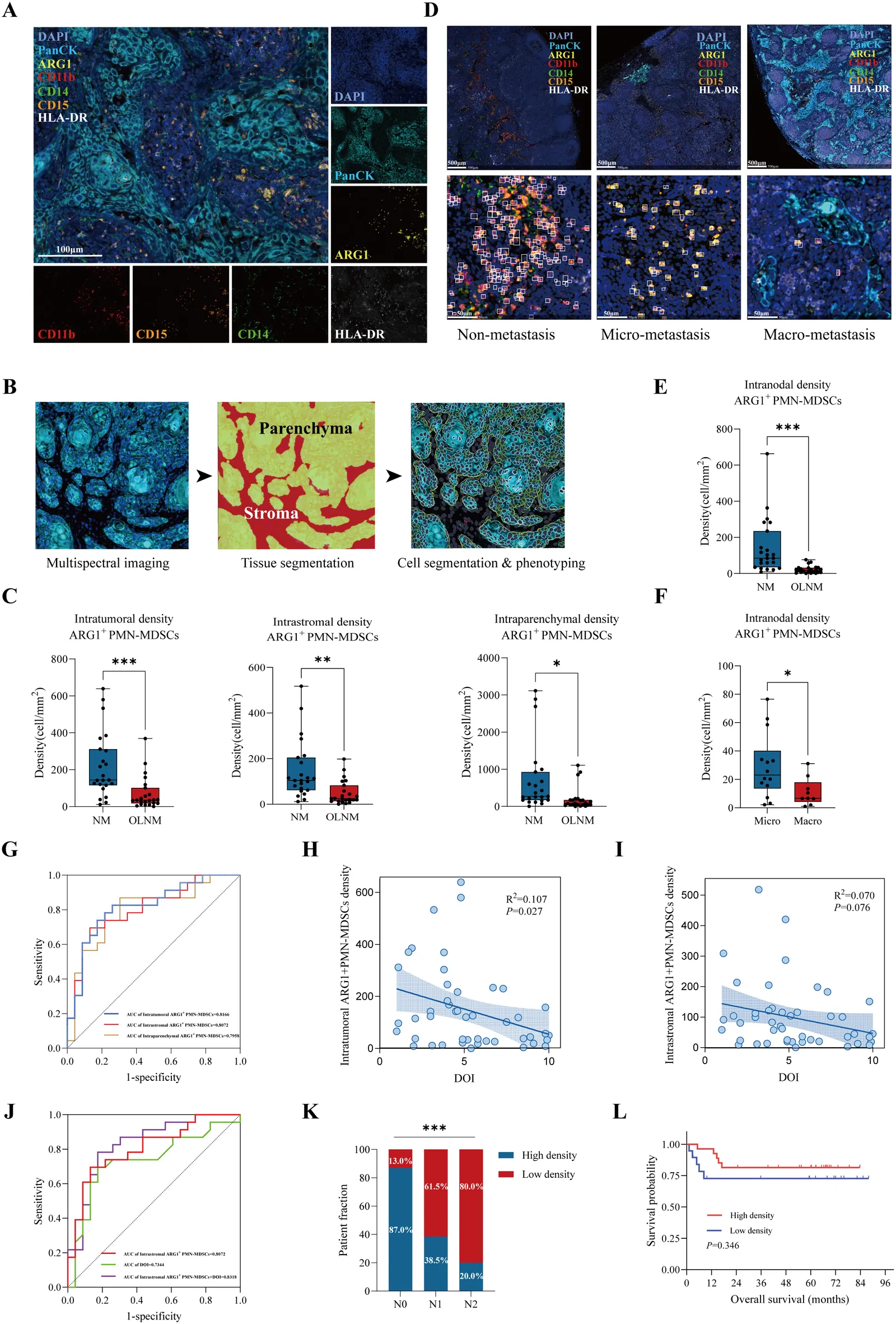
Spatial ARG1+ PMN-MDSC density enhances OLNM risk stratification. **(A)** Representative multiplex immunofluorescence (mIF) images. Scale bar, 100μm. **(B)** HALO-based spatial segmentation classifying regions of interest into tumor parenchyma and stroma based on PanCK expression. **(C)** Comparison of ARG1^+^ PMN-MDSC density between the NM and OLNM groups across different spatial compartments (Total Tumor, Stroma, and Parenchyma) in primary lesions. **(D)** Representative mIF images of ARG1^+^ PMN-MDSCs in paired cervical lymph nodes. Scale bar, 500μm(top), 50μm(bottom). **(E)** Quantification of ARG1^+^ PMN-MDSCs in paired cervical lymph nodes. **(F)** Density of ARG1^+^ PMN-MDSCs in metastatic lymph nodes stratified by tumor burden (micro- vs. macro-metastasis).**(G)** Receiver operating characteristic (ROC) curves evaluating the diagnostic performance of spatial ARG1^+^ PMN-MDSC densities. **(H, I)** Pearson correlation analysis between depth of invasion (DOI) and total tumor **(H)** or intrastromal **(I)** ARG1^+^ PMN-MDSC densities. **(J)** ROC curves comparing the predictive accuracy of DOI alone versus a combined model integrating DOI with intrastromal density. **(K)** Clinical relevance of intrastromal ARG1^+^ PMN-MDSC infiltration across different clinical N stages. Patients were stratified into high- and low-density groups based on the ROC-derived cutoff value, with 43.48% of patients classified as low-density. **(L)** Kaplan-Meier survival curves for overall survival (OS) stratified by intrastromal ARG1^+^ PMN-MDSC density. Data are presented as mean ± SD. Statistical analysis was performed using unpaired Student’s t-test for comparison between groups, and Fisher’s exact test for categorical data. *P < 0.05, **P < 0.01, ***P < 0.001. NM, non-metastasis; OLNM, occult lymph node metastasis; mIF, multiplex immunofluorescence; ARG1, arginase-1; PanCK, pan-cytokeratin; PMN-MDSC, polymorphonuclear myeloid-derived suppressor cell; DOI, depth of invasion; ROC, receiver operating characteristic; OS, overall survival.

To validate the proteomic findings and characterize the distribution of ARG1-expressing cells across different tissue regions, we established a validation cohort comprising 23 matched pairs (46 samples) of primary TSCC tissues and paired cervical lymph nodes. Multiplex immunofluorescence was performed using a six-marker panel (CD11b, CD15, ARG1, CD14, HLA-DR, PanCK) (**Figure 2A**). Based on PanCK expression, the HALO supervised image analysis system classified regions of interest (ROIs) into tumor parenchyma and stromal compartments (**Figure 2B**). Phenotypic profiling identified PMN-MDSCs (CD11b^+^CD15^+^ARG1^+^CD14^-^HLA-DR^-^) as the predominant ARG1^+^ population (**Supplementary Figure 2D**). To determine whether variation in ARG1^+^ PMN-MDSC abundance was attributable to altered ARG1 expression within PMN-MDSCs, we quantified the proportion of ARG1-expressing cells among phenotypically defined PMN-MDSCs (CD11b^+^CD15^+^CD14^-^HLA-DR^-^). The proportion of ARG1-expressing cells was highly comparable between NM and OLNM groups (92.54% vs. 92.13%) (**Supplementary Figure 2E**), indicating that ARG1 expression within this population remained consistent regardless of metastatic status. These findings suggest that the difference in ARG1^+^ PMN-MDSC abundance between NM and OLNM groups was unlikely to result from selective loss of ARG1 expression, but rather reflected variation in the abundance of this ARG1-expressing population.

#### 3.3.2 Consistent depletion of ARG1^+^ PMN-MDSCs in primary and metastatic sites

Quantitative analysis revealed a significant reduction in ARG1^+^ PMN-MDSC density (cells/mm^2^) in the OLNM group compared to the NM group. In primary lesions, this reduction was profoundly significant within the total tumor area (Parenchyma + Stroma) (*P* < 0.001), driven by a highly significant reduction in the stroma (*P* < 0.001) and a notable decrease within the tumor parenchyma (*P* < 0.05) (**Figure 2C**). To determine if this immunological pattern persists in the lymphatic niche, we analyzed the paired cervical lymph nodes. Consistent with the primary tumor findings, lymph nodes from the OLNM group exhibited significantly lower ARG1^+^ PMN-MDSC densities than those from the NM group (*P<*0.001) (**Figures 2D, E**). Furthermore, we observed a progressive loss of these cells associated with metastatic burden: lymph nodes with pathological macro-metastasis (>2 mm) displayed significantly lower ARG1^+^ PMN-MDSC densities compared to those with micro-metastasis (<2 mm) (*P* = 0.035) (**Figure 2F**). This downward gradient—from non-metastatic to macro-metastatic states—corroborates the association between the loss of intracellular ARG1 and metastatic progression.

#### 3.3.3 Diagnostic performance and synergy with DOI

We next evaluated the predictive value of ARG1^+^ PMN-MDSC density. ROC curve analysis demonstrated that parenchymal and stromal densities achieved AUC values of 0.7958 and 0.8072, respectively. The integrated intratumoral density (combined parenchyma and stroma) yielded the highest discriminative accuracy (AUC = 0.8166) (**Figure 2G**). Further analysis integrated these immune features with DOI, a standard pathological predictor. In our cohort, DOI was significantly higher in OLNM patients (*P* < 0.01), achieving an independent AUC of 0.7344 (**Figure 2J**). Correlation analysis revealed that while total intratumoral ARG1^+^ PMN-MDSC density showed a weak positive correlation with DOI (R^2^ = 0.107, *P =* 0.027) (**Figure 2H**), intrastromal density exhibited no significant correlation (R^2^ = 0.070, *P =* 0.076) (**Figure 2I**). The lack of significant correlation between stromal ARG1^+^ PMN-MDSC density and DOI suggests that this immune feature may capture biological information complementary to tumor invasion depth. The combined model incorporating DOI and intrastromal ARG1^+^ PMN-MDSC density demonstrated improved discrimination compared with DOI alone, with an AUC of 0.8318 (**Figure 2J**).

#### 3.3.4 Clinical relevance

Finally, we assessed the association between ARG1^+^ PMN-MDSC density and clinical metrics. To stratify patients according to intrastromal ARG1^+^ PMN-MDSC density, the optimal cutoff value was objectively determined by maximizing the Youden Index from the ROC curve, with OLNM status used as the outcome variable. Patients with density values below this ROC-derived cutoff were classified into the low-density group, whereas those above the cutoff were classified into the high-density group. This ROC-derived threshold resulted in 43.48% of patients being categorized as the low-density group, thereby reducing investigator-defined grouping bias. Based on this stratification, we found that lower intrastromal infiltration was associated with advanced N stage (**Figure 2K**), but not with other features such as T stage, DOI, recurrence, differentiation, or growth patterns (all *P* > 0.05) (**Supplementary Figures 2F–J**). Survival analysis indicated a trend toward poorer OS in patients with lower infiltration, although statistical significance was not reached in this cohort (*P* = 0.346) (**Figure 2L**).

### 3.4 Proximity analysis of ARG1^+^ PMN-MDSCs relative to tumor cells

#### 3.4.1 Increased intercellular proximity distance in OLNM

**Figure 3:**
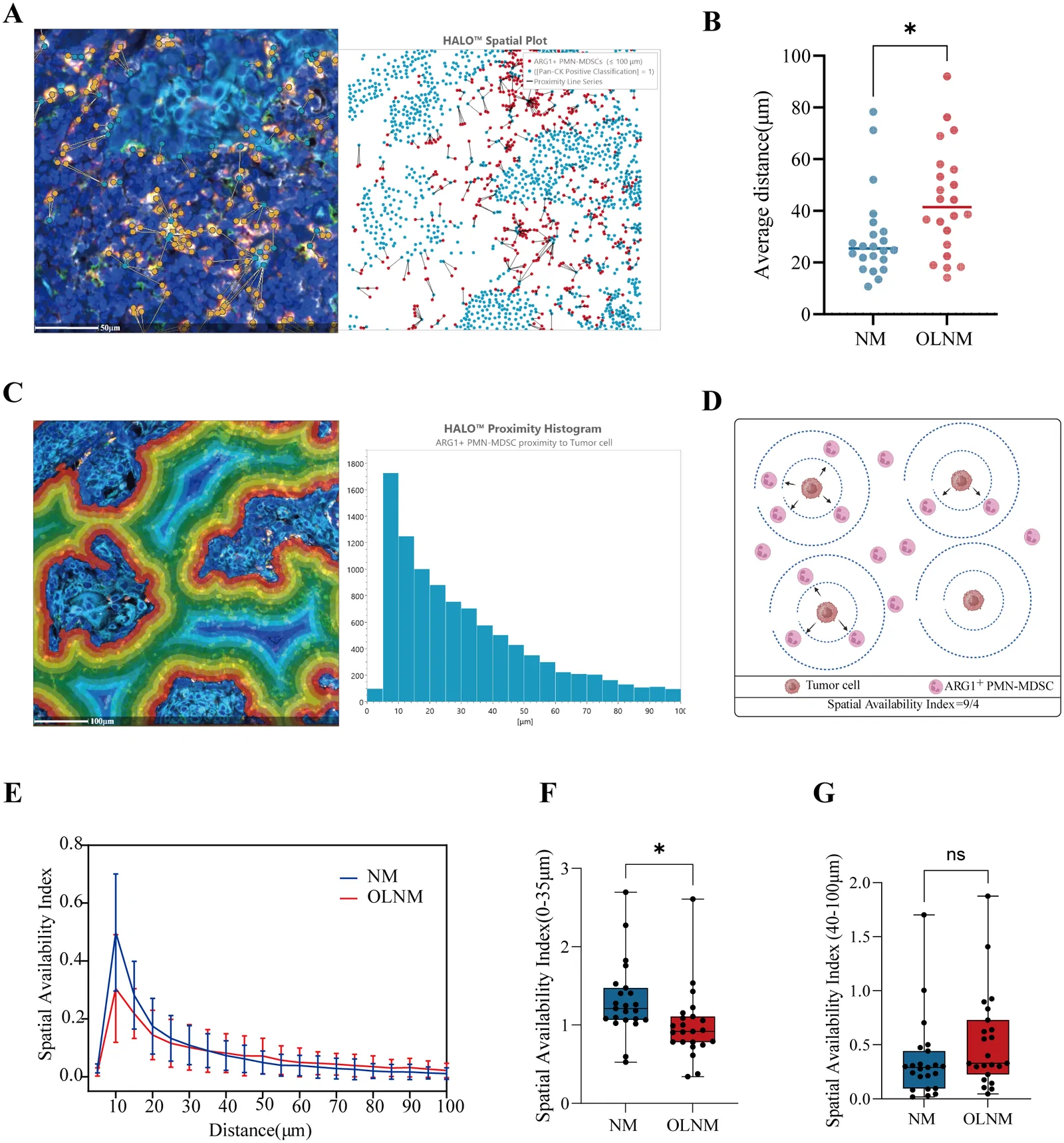
Spatial proximity analysis reveals localized depletion of ARG1+ PMN-MDSCs at the tumor-stroma interface. **(A)** Representative visualization of proximity analysis using the HALO platform, measuring the distance from ARG1+ PMN-MDSCs to the nearest PanCK+ tumor cells. Scale bar, 50μm. **(B)** Comparison of the mean distance from ARG1+ PMN-MDSCs to the nearest tumor cell between the NM and OLNM groups. **(C)** Absolute abundance of ARG1+ PMN-MDSCs at 5 μm intervals (0–100μm) from the tumor boundary. Scale bar, 100μm. **(D)** Schematic illustration of the “Spatial Availability Index” calculation, defined as the ARG1+ PMN-MDSC count normalized to the number of tumor cells within corresponding distance intervals. Created with https://www.BioRender.com. **(E)** Segmental analysis of the Spatial Availability Index identifying divergent patterns in the proximal (0–35μm) and distal (40–100μm) zones. **(F)** Comparison of the Spatial Availability Index in the proximal segment (0–35μm). **(G)** Comparison of the Spatial Availability Index in the distal segment (40–100μm). Data are presented as mean ± SD. Statistical analysis was performed using unpaired Student’s t-test for distance and segmental comparisons, and two-way ANOVA followed by Sidak’s multiple comparisons test for the distribution data in **(E)**. *P < 0.05, **P < 0.01, ***P < 0.001. Abbreviations: NM, non-metastasis; OLNM, occult lymph node metastasis; PanCK, pan-cytokeratin; ARG1, arginase-1; PMN-MDSC, polymorphonuclear myeloid-derived suppressor cell.

To quantify the spatial relationship between ARG1^+^ PMN-MDSCs and tumor cells, we performed a proximity analysis using the HALO platform (**Figure 3A**). In the OLNM group, the mean distance between ARG1^+^ PMN-MDSCs and the nearest PanCK^+^ tumor cell was significantly greater than in the NM group (43.76 ± 20.84μm vs 30.02 ± 17.07μm; *P* = 0.0213) (**Figure 3B**). This indicates that ARG1^+^ PMN-MDSCs in patients with occult metastasis are located further from the primary tumor front compared to those in non-metastatic cases.

#### 3.4.2 Evaluation of the “spatial availability index”

We analyzed the absolute abundance of ARG1^+^ PMN-MDSCs at 5μm intervals (from 0 to 100μm) from the tumor cells. While cellular abundance generally followed a downward gradient with increasing distance in both groups (**Figure 3C**), we observed distinct distribution patterns. To account for variations in tumor cell density across samples, we established a “Spatial Availability Index”, defined as the abundance of ARG1^+^ PMN-MDSCs within a specific distance normalized to the number of tumor cells in the corresponding region (**Figure 3D**). Segmental analysis of this index identified two distinct patterns associated with metastatic status (**Figure 3E**): Proximal Segment (0–35μm): Within 35μm of the tumor edge, the NM group exhibited a significantly higher Spatial Availability Index compared to the OLNM group (*P* = 0.0225) (**Figure 3F**). The most pronounced differences were localized at the 5–10μm (*P* < 0.001) and 10–15μm (*P* < 0.05) intervals. Distal Segment (40–100μm): In contrast, no significant difference in Spatial Availability Index was observed between the NM and OLNM groups within the 40–100 μm range (P = 0.1634) (**Figure 3G**). These findings indicate that the reduction of ARG1^+^ PMN-MDSC availability in OLNM cases is primarily localized at the immediate tumor–stroma interface rather than extending throughout the distal stromal compartment. This spatially restricted alteration suggests that impaired ARG1^+^ PMN-MDSC enrichment near the tumor boundary may represent a key feature associated with occult lymph node metastasis.

### 3.5 Spatial inversion of ARG1^+^ PMN-MDSCs and NETosis at the 35μm peritumoral hotspot

The localized reduction of intact ARG1^+^ PMN-MDSCs at the immediate tumor-stroma interface raised the possibility of functional remodeling of the myeloid compartment and prompted us to investigate its spatial relationship with NET-associated signals. This hypothesis was supported by our bioinformatic analysis, which revealed the significant enrichment of GO and KEGG pathways representing key mediators of NETosis, including the extracellular space, CXCR binding, NF-kappa B and Toll-like receptor signaling (**Figures 1D, E**). To investigate this potential link, we performed an expanded spatial mIF analysis using a refined six-marker panel (CD11b, CD15, ARG1, CitH3, MPO, PanCK).

**Figure 4:**
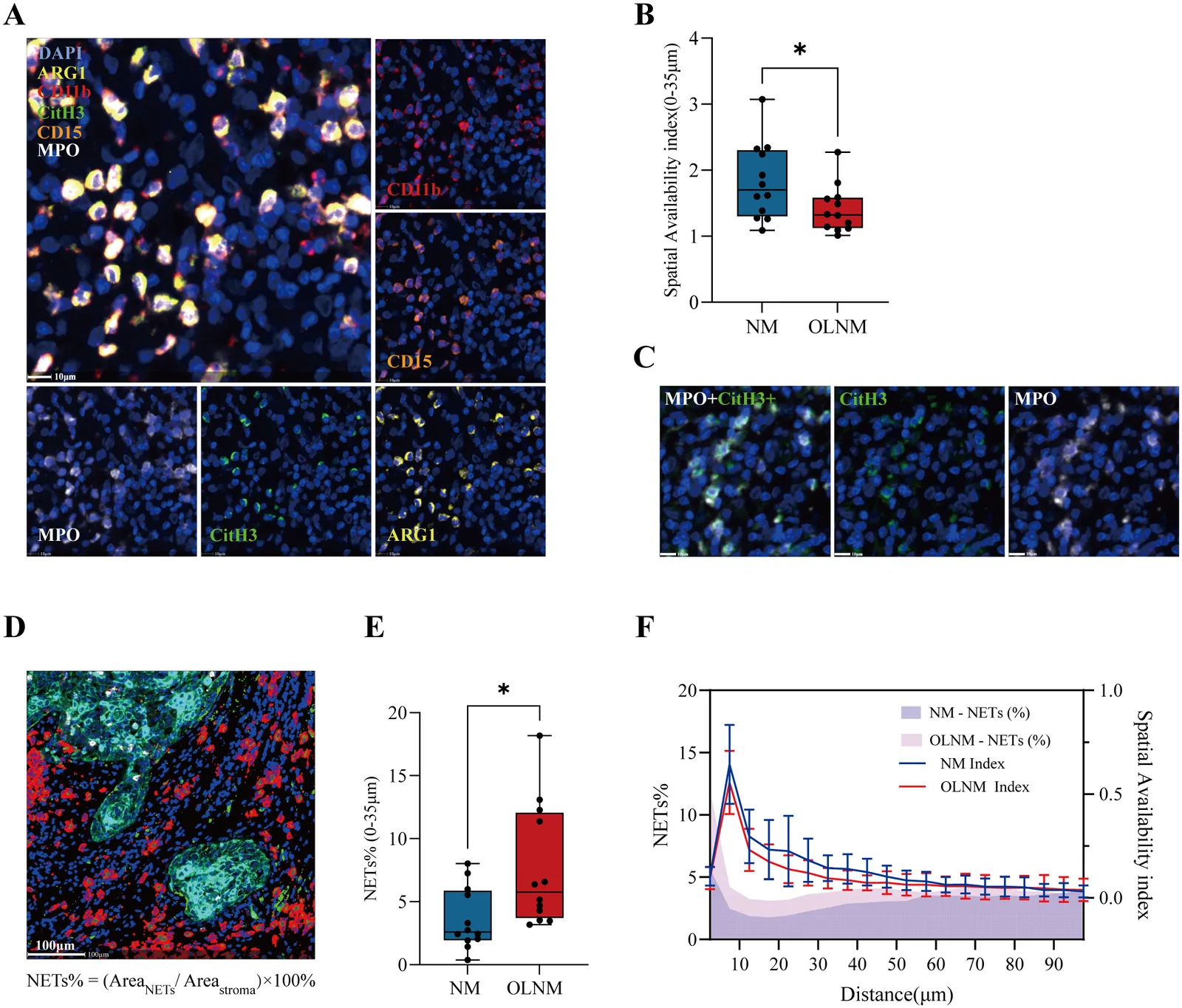
Spatial inversion between intact ARG1+ PMN-MDSCs and NETosis within the 35 μm peritumoral hotspot. **(A)** Representative mIF images of CD11b^+^CD15^+^ARG1^+^ PMN-MDSCs co-expressing CitH3 and MPO. Scale bar, 10μm. **(B)** Spatial Availability Index of intact ARG1^+^ PMN-MDSCs within the 0–35 μm proximal segment. **(C)** Representative mIF images demonstrating CitH3^+^MPO^+^ double-positive NETs. Scale bar, 10μm. **(D)** Schematic illustration of the NETs% calculation, defined as the percentage of NETs area relative to the stromal area. Scale bar, 100μm. **(E)** Comparison of NETs% infiltration within the 0–35 μm peritumoral range between groups. **(F)** Double Y-axis plot illustrating the reciprocal spatial relationship between NETs% (left) and the Spatial Availab ility Index of ARG1+ PMN-MDSCs (right) across distance intervals. Data are presented as mean ± SD. Statistical analysis was performed using unpaired Student’s t-test for comparison between groups, and two-way ANOVA followed by Sidak’s multiple comparisons test for the spatial relationship data in **(F)**. *P < 0.05, **P < 0.01, ***P < 0.001. Abbreviations: NM, non-metastasis; OLNM, occult lymph node metastasis; PMN-MDSC, polymorphonuclear myeloid-derived suppressor cell; MPO, myeloperoxidase; CitH3, citrullinated histone H3; mIF, multiplex immunofluorescence; NETs, neutrophil extracellular traps; NETosis, neutrophil extracellular trap formation (the process of NET release).

*In situ* analysis confirmed the spatial association between NETs-associated signals and the ARG1^+^ population. Specifically, we identified CD11b^+^CD15^+^ARG1^+^ PMN-MDSCs exhibiting high co-expression of the NETosis markers MPO and CitH3 (**Figure 4A**).

Focusing on the previously identified 0-35μm “Proximal Segment,” we observed a striking spatial inversion between two myeloid states: intact ARG1^+^ PMN-MDSCs and CitH3^+^MPO^+^ NETs. Within this 35μm peritumoral hotspot, the Spatial Availability Index of intact ARG1^+^ PMN-MDSCs remained significantly higher in the NM group compared to the OLNM group (*P* = 0.047) (**Figures 4B, F**), corroborating our initial findings of ARG1^+^ cellular reduction in metastatic cases. Conversely, the accumulation of NETs% — defined by CitH3+MPO+ double-positive areas (**Figure 4C**) and quantified as the percentage of NETs area relative to the stromal area (**Figure 4D**) — exhibited the opposite trend, with the OLNM group displaying significantly higher NETs infiltration within the same proximal range (*P* = 0.017) (**Figures 4E, F**).

This reciprocal distribution suggests that in patients with occult metastasis, reduction of intact ARG1^+^ PMN-MDSCs is accompanied by increased NET-associated signals at the tumor front, indicating spatial remodeling of the myeloid compartment. This spatial reorganization defines a specialized “hotspot” at the 35μm interface, potentially serving as a physical and biochemical driver of early lymphatic dissemination.

## 4 Discussion

The clinical management of the neck in early-stage (cT1-2N0) TSCC remains hampered by a lack of high-resolution biomarkers capable of accurately predicting occult lymph node metastasis. In this study, we addressed this critical gap by mapping the spatiotemporal and proteomic dynamics of the TSCC immune microenvironment. Moving beyond traditional cellular enumeration, we uncovered a paradoxical “Myeloid Explosion” phenomenon characterized by the localized spatial reduction of ARG1^+^ PMN-MDSCs and the reciprocal accumulation of neutrophil extracellular traps at the immediate tumor-stroma interface. By integrating these spatial immune signatures with standard pathological metrics (DOI), we developed a spatio-clinical model (AUC = 0.8318) that outperforms traditional staging, offering a potential framework for identifying patients at higher risk of occult lymph node metastasis who may benefit from personalized neck management strategies after further clinical validation.

A pivotal yet counterintuitive proteomic signature was the “Tissue-Low, Serum-High” discrepancy of ARG1 and IL-8 in patients with occult metastasis. Traditionally, the accumulation of PMN-MDSCs within the tumor stroma is viewed as a primary driver of local immune suppression (20, 32). However, our integrated spatial analysis provides a potential explanation for this apparent discrepancy. We demonstrate that within the 0–35μm “Proximal Segment,” ARG1^+^ PMN-MDSC reduction is spatially accompanied by increased NET-associated signals, suggesting localized remodeling of the myeloid compartment at the tumor–stroma interface. This spatial pattern suggests that NET-associated remodeling may be accompanied by a reduction of intact ARG1^+^ PMN-MDSCs within the primary tumor stroma and paired lymph nodes, although the direct cellular relationship between these events requires further validation. Given the independent tissue and serum cohorts, we cannot determine whether reduced intratumoral ARG1 levels directly contribute to elevated circulating ARG1. Nevertheless, the divergent tissue and serum ARG1 profiles indicate a compartment-specific alteration of ARG1 distribution associated with occult metastasis. These findings may contribute to a systemic immunosuppressive state, effectively “priming” the pre-metastatic niche in the regional lymph nodes before physical tumor cell dissemination occurs (19, 33–36). Indeed, this divergent expression pattern strongly corroborates recent findings in head and neck cancers, which demonstrated that while ARG1 levels are reduced within the primary tumor tissues, there is a paradoxical surge of plasma-derived exosomal ARG1 associated with metastasis (37).

Furthermore, our proximity analysis revealed a significant spatial reduction of intact PMN-MDSCs in metastatic cases. In the OLNM group, intact ARG1^+^ cells were located significantly further from the tumor cells, while depleting in the proximal zone. Consistent with recent reports that the spatial displacement of MDSCs towards non-tumor areas drives immune suppression and poorer survival in OSCC (38), our data connect these phenomena: the distal spatial retention of intact MDSCs and the systemic surge of ARG1 are, in fact, complementary outcomes of the localized terminal NETosis occurring at the proximal invasion front.

This spatial heterogeneity suggests that the tumor-stroma interface acts as a highly reactive zone, closely correlated with the dynamic partitioning of microenvironmental chemokines such as IL-8 and CXCL1, which exhibited divergent tissue-versus-serum profiles in close concordance with the expression trends of ARG1 in our Olink data. These chemokines not only recruit myeloid cells but are potent inducers of NETosis (27, 39–41). NET-associated structures at this spatial boundary may contribute to an immunologically restrictive microenvironment. Recent high-impact studies have demonstrated that NETs can coat tumor cells, shield them from cytotoxicity, and physically restrict the infiltration of CD8^+^ T cells and NK cells into the tumor parenchyma (27, 42, 43). Thus, localized NET-associated remodeling at the invasion front may contribute to an immunosuppressive niche that facilitates tumor dissemination, although the functional consequences on adaptive immune cell infiltration require further investigation.

From a clinical perspective, our findings highlight the inherent limitations of relying solely on morphological parameters for risk stratification. While current guidelines often use a DOI threshold of 4 mm to recommend END, our cohort demonstrated that 18.18% of patients in this “low-risk” category still harbored occult metastasis. The lack of significant correlation between intrastromal ARG1^+^ PMN-MDSC density and DOI suggests that immune remodeling may provide complementary information beyond conventional measures of tumor invasion. Our combined spatio-clinical model provides a biologically informed approach for improving OLNM risk stratification beyond conventional pathological parameters. Additionally, given that the efficacy of immune checkpoint blockade (ICB) in head and neck cancers is often severely limited by dense myeloid suppressive networks (44, 45), targeting the formation of NETs could emerge as a novel therapeutic strategy to intercept early lymphatic dissemination (27, 46, 47). The identification of a spatially restricted NET-associated barrier within the 35 μm tumor–stroma interface highlights a potential therapeutic window for targeting early metastatic remodeling. Disrupting this localized NET-associated architecture may reshape the tumor microenvironment. However, whether such intervention restores effective antitumor immunity or enhances responses to immune checkpoint blockade remains to be determined.

Despite these promising insights, several limitations remain. First, although ARG1^+^ PMN-MDSCs were identified based on established human PMN-MDSC phenotypic criteria, we acknowledge that these cells share considerable phenotypic overlap with conventional neutrophils. Functional immunosuppressive assays were not performed in the present study, and further studies are required to validate their suppressive activity and biological functions. Second, our findings and predictive model derived from a single retrospective cohort require validation in independent, large-scale multicenter studies to ensure global reproducibility. Third, while our data clearly define the spatial overlap of these events, further functional *in vitro* and *in vivo* verification is warranted to fully elucidate the complex microenvironmental crosstalk among IL-8 signaling, NETosis, and ARG1 release. Future studies should determine whether ARG1^+^ PMN-MDSC assessment can be reliably performed in preoperative biopsy specimens and whether biopsy-based measurements accurately reflect the immune landscape of the entire tumor. Prospective validation in larger cohorts will be required before this biomarker can be incorporated into clinical algorithms for elective neck management. In parallel, circulating ARG1 and IL-8 may represent complementary minimally invasive biomarkers for monitoring systemic immune alterations associated with occult metastasis.

In conclusion, this study maps the spatial architecture of the immune microenvironment in early-stage TSCC, highlighting a potential link between PMN-MDSC alterations, NET-associated remodeling, and metastatic risk. These spatial insights not only refine our understanding of tumor-immune co-evolution but also offer a potential immune-based framework for personalized risk stratification in early-stage tongue cancer.

## Data Availability

The datasets presented in this study can be found in online repositories. The names of the repository/repositories and accession number(s) can be found in the article/**Supplementary Material**.
