# Supplementary material for: The spatial landscape of the peritumoral interface: ARG1+ PMN-MDSCs reduction and NETosis as indicators of occult metastasis in tongue squamous cell carcinoma
Source: Front Immunol. 2026 Aug 19;17:1910935. doi: 10.3389/fimmu.2026.1910935 (PMC13534108; doi:10.3389/fimmu.2026.1910935)
Supplement: Supplementary file 1 [file Table1.doc]

**Supplementary Figure Legend**

**Figure S1. Overview of patient enrollment, specimen availability, and experimental workflow.**

A retrospective tissue cohort comprising 113 patients with early-stage TSCC was established. Fresh-frozen tumor tissues available from 55 patients were used for tumor tissue Olink proteomic profiling after propensity score matching (PSM), resulting in 17 matched pairs (n = 34). Formalin-fixed paraffin-embedded (FFPE) primary tumors and corresponding cervical lymph nodes were available from the retrospective tissue cohort and were subjected to independent PSM, yielding 23 matched pairs (n = 46) for multiplex immunofluorescence (mIF) and digital spatial analysis. A subset of 24 primary tumor sections was subsequently selected for NETosis validation. In addition, an independent serum cohort (n = 17) was prospectively collected for preoperative serum Olink proteomic profiling.

**Figure S2. Proteomic screening and clinical characterization of ARG1^+^ PMN-MDSCs.**

**(A)** Heatmap of differentially expressed proteins (DEPs) in primary tumor tissues between the NM and OLNM groups.

**(B)** Heatmap of DEPs in preoperative serum.

**(C)** AUC-based ranking of the top 5 diagnostic markers for OLNM prediction.

**(D)** Proportional analysis of PMN-MDSCs (CD11b^+^CD15^+^ARG1^+^CD14^-^HLA-DR^-^) within the total ARG1^+^ population.

**(E)** Proportion of ARG1-expressing cells among phenotypically defined PMN-MDSCs

**(F–J)** Association between intrastromal ARG1^+^ PMN-MDSC density (High vs. Low) and clinical parameters, including T stage (F), depth of invasion (G), recurrence (H), differentiation (I), and growth patterns (J).

Statistical analysis was performed using Fisher’s exact test for categorical associations in (F–J). **P* < 0.05, ***P* < 0.01, ****P* < 0.001. Abbreviations: NM, non-metastasis; OLNM, occult lymph node metastasis; ARG1, arginase-1; PMN-MDSC, polymorphonuclear myeloid-derived suppressor cell; HLA-DR, human leukocyte antigen-DR; AUC, area under the curve; DOI, depth of invasion.
