# Supplementary material for: The spatial landscape of the peritumoral interface: ARG1+ PMN-MDSCs reduction and NETosis as indicators of occult metastasis in tongue squamous cell carcinoma
Source: Front Immunol. 2026 Aug 19;17:1910935. doi: 10.3389/fimmu.2026.1910935 (PMC13534108; doi:10.3389/fimmu.2026.1910935)
Supplement: Supplementary file 2 [file Table2.doc]

| **Table S1 \| Baseline characteristics of the Olink cohort (after PSM)** | | | |
| --- | --- | --- | --- |
| **Characteristics** | **OLNM group** | **NM group** | ***P*** |
|  | **Olink cohort (after PSM)** | |  |
|  | n=17 | n=17 |  |
| **Age (mean ± SD)** | 60.00±13.15 | 54.47±11.41 | 0.200 |
| **Gender** |  |  | >0.999 |
| Male | 12(70.59%) | 12(70.59%) |  |
| Female | 5(29.41%) | 5(29.41%) |  |
| **Smoking history** |  |  | 0.296 |
| Yes | 5(29.41%) | 9(52.94%) |  |
| No | 12(70.59%) | 8(47.06%) |  |
| **Alcohol history** |  |  | >0.999 |
| Yes | 6(35.29%) | 7(41.18%) |  |
| No | 11(64.71%) | 10(58.82%) |  |
| **cT grade** |  |  | 0.085 |
| T1 | 1(5.88%) | 6(35.29%) |  |
| T2 | 16(94.12%) | 11(64.71%) |  |
| **DOI (mm)** | 5.60±2.74 | 4.49±2.24 | 0.089 |
| **Differentiation** |  |  | >0.999 |
| Well | 4(23.53%) | 4(23.53%) |  |
| Moderate | 9(52.94%) | 10(58.82%) |  |
| Poor | 4(23.53%) | 3(17.65%) |  |
| **Growth pattern** |  |  | 0.141 |
| Exophytic | 1(5.89%) | 5(29.41%) |  |
| Infiltrative | 12(70.59%) | 7(41.18%) |  |
| Ulcerative | 4(23.53%) | 5(29.41%) |  |

Data are shown as mean±SD or n (%). Continuous variables were compared using unpaired t-tests, and categorical variables using Chi-square or Fisher’s exact tests. *P < 0.05, **P < 0.01, ***P < 0.001. Abbreviations: NM, non-metastasis; OLNM, occult lymph node metastasis; DOI, depth of invasion.
