# Supplementary material for: The spatial landscape of the peritumoral interface: ARG1+ PMN-MDSCs reduction and NETosis as indicators of occult metastasis in tongue squamous cell carcinoma
Source: Front Immunol. 2026 Aug 19;17:1910935. doi: 10.3389/fimmu.2026.1910935 (PMC13534108; doi:10.3389/fimmu.2026.1910935)
Supplement: Supplementary file 3 [file Table3.doc]

| **Table S2 \| Baseline characteristics of the mIF cohort (after PSM)** | | | |
| --- | --- | --- | --- |
| **Characteristics** | **OLNM group** | **NM group** | ***P*** |
|  | **mIF cohort (after PSM)** | |  |
|  | n=23 | n=23 |  |
| **Age (mean ± SD)** | 52.78±11.09 | 51.22±11.47 | 0.640 |
| **Gender** |  |  | >0.999 |
| Male | 19(82.61%) | 19(82.61%) |  |
| Female | 4(17.39%) | 4(17.39%) |  |
| **Smoking history** |  |  | 0.768 |
| Yes | 10(43.48%) | 12(52.17%) |  |
| No | 13(56.52%) | 11(47.83%) |  |
| **Alcohol history** |  |  | >0.999 |
| Yes | 9(39.13%) | 8(34.78%) |  |
| No | 14(60.87%) | 15(65.22%) |  |
| **cT grade** |  |  | >0.999 |
| T1 | 1(4.35%) | 1(4.35%) |  |
| T2 | 22(95.65%) | 22(95.65%) |  |
| **DOI (mm)** | 6.20±2.71 | 4.20±2.20 | 0.006** |
| **Differentiation** |  |  | 0.625 |
| Well | 5(21.74%) | 5(21.74%) |  |
| Moderate | 11(47.83%) | 14(60.87%) |  |
| Poor | 7(30.43%) | 4(17.39%) |  |
| **Growth pattern** |  |  | 0.342 |
| Exophytic | 2(8.70%) | 6(26.09%) |  |
| Infiltrative | 14(60.87%) | 12(52.17%) |  |
| Ulcerative | 7(30.43%) | 5(21.74%) |  |

Data are shown as mean±SD or n (%). Continuous variables were compared using unpaired t-tests, and categorical variables using Chi-square or Fisher’s exact tests. *P < 0.05, **P < 0.01, ***P < 0.001. Abbreviations: NM, non-metastasis; OLNM, occult lymph node metastasis; DOI, depth of invasion.
